# Are there socio-demographic differences in salt behaviours and fruit and vegetable consumption in Australian adults? A nationally representative cross-sectional survey

**DOI:** 10.1186/s12937-021-00734-0

**Published:** 2021-09-08

**Authors:** Emalie Rosewarne, Joseph Alvin Santos, Annet Hoek, Carley Grimes, Caryl Nowson, Jacqui Webster, Kristy A. Bolton

**Affiliations:** 1 The George Institute for Global Health, The University of New South Wales, Sydney, 2006 Australia; 2 Institute for Physical Activity and Nutrition, School of Exercise and Nutrition Science, Deakin University, Geelong, 3220 Australia

**Keywords:** Salt, Fruit, Vegetables, Socio-demographic factors, Australia

## Abstract

**Background:**

Diets low in fruit and vegetables and high in salt are among the top dietary risk factors for non-communicable diseases (NCDs). Using a nationally representative sample of Australians, this study aimed to describe self-reported intake of fruit and vegetables, and knowledge, attitudes and behaviours related to salt intake, and determine if there were socio-demographic differences between population subgroups.

**Methods:**

A 2016 cross-sectional survey of Australian adults aged 18 years and over, which comprised 160 questions, including socio-demographic and health-related questions. Descriptive statistics (mean, 95% confidence interval, %) were calculated. Weighted-adjusted logistic regression models were used to determine if there were socio-demographic differences in salt behaviours and fruit and vegetable consumption.

**Results:**

A total of 1217 participants completed the survey (51% female). Less than 8% of participants reported consuming the recommended 2 or more serves of fruit and 5 or more serves of vegetables. Almost 60% of participants frequently added salt during cooking/meal preparation and 42% of respondents frequently placed a salt-shaker on the table at mealtimes. There were no consistent patterns between socio-demographic factors and measures of fruit and vegetable consumption and salt behaviours. Differences in at least one measure were found for sex, age, location, education level and weight category.

**Conclusions:**

There were no consistent patterns between socio-demographic factors and salt behaviours and fruit and vegetable intake. Less than recommended intakes of fruit and vegetables and frequent discretionary salt use are placing Australians at risk of diet-related NCDs. Broad population-based policies and programs to improve fruit and vegetable intake and salt behaviours are needed to improve Australian’s diets.

**Supplementary Information:**

The online version contains supplementary material available at 10.1186/s12937-021-00734-0.

## Introduction

Australian diets are nutritionally imbalanced, with diets low in fruit and vegetables and high in salt among the top risk factors (1). It is estimated that only 5% of Australians are meeting the recommended guideline of 2 or more serves of fruit and 5 or more serves of vegetables per day, with just over half of Australian adults meeting the fruit guideline alone and only 7.5% meeting the vegetable guideline alone (2). For salt, Australians are consuming almost double (3) the recommended daily maximum amount of 5 g (4), placing them at increased risk of developing high blood pressure (5). Low consumption of fruit and vegetables, and high salt intakes, increase population risk of cardiovascular diseases (CVDs), diabetes, kidney disease and some cancers (1). Together, these three dietary risk factors account for more than 11,500 deaths and almost 192,000 disability-adjusted life years in Australia each year (1).

Strategies to improve Australian diets, by increasing fruit and vegetable intake and decreasing salt intake, offer an opportunity to reduce Australia’s disease burden. Strategies should include interventions to improve the food environment, through food reformulation and nutrition standards in public settings (6, 7), supplemented by programs to raise consumer awareness and positively shift consumer behaviours (8). The consumer strategy should be informed by a comprehensive understanding of the population’s knowledge, attitudes and behaviours (KABs) relating to healthy diets, including fruit, vegetable and salt intakes (9). This includes whether KABs differ by population subgroups such as sex, age, location and socioeconomic background, and if specific strategies are needed to reach different groups.

Self-reported fruit and vegetable intake data are routinely collected in national health surveys, however, these data are only disaggregated by age and sex (2, 10, 11). One study in young adults aged 18–34 years determined fruit and vegetable intake differed by selected socio-demographic factors (e.g. sex, age, location) (12). It is possible these differences exist across all age groups. Another study found no difference in fruit and vegetable score by levels of socioeconomic disadvantage, education level or household income when using a dietary guidelines index (13). Given the varying results in past studies, further research to determine if there are socio-demographic differences in fruit and vegetable intakes in Australia is necessary.

Several studies have investigated salt-related KABs in Australia. However, most surveys have not been administered to a nationally representative sample (14–21), and many have only included questions about two discretionary salt behaviours, adding salt at the table or during cooking/meal preparation (15–17, 21). The most recent national salt-related KAB survey, which was conducted in 2007 and comprised 23 questions, was one of only a few studies that have asked a more extensive set of questions about salt-related KABs and the only comprehensive survey in a nationally representative sample (22). Given the time elapsed since the last nationally representative survey, an update on current salt-related KABs is warranted and would be useful in informing a future national consumer campaign. When comparing the results from more recent subnational studies (14–21), large variations in participant responses were evident, likely due to the differences in sample characteristics and sampling methods. For example, the proportion of participants frequently adding salt during meal preparation varied from 46% (19) to 84% (21) and at the table from 36% (16) to 68% (14). Given the variability in participant responses between subnational studies, differences in salt-related KABs between population subgroups should be considered. In a large Victorian study, differences in salt-related KABs were identified by socio-demographic factors, including sex, age and socioeconomic status (18), and this may be true across Australia, though no study has yet investigated this.

In a nationally representative sample of Australians, this study aimed to describe self-reported intake of fruit and vegetables and knowledge, attitudes and behaviours related to salt intake, and determine if these measures differed between socio-demographic population subgroups (i.e. sex, age, location, educational attainment, language, weight category and area-level disadvantage).

## Methods

### Participant recruitment

This was a nationally-representative cross-sectional survey of Australian adults aged 18 years and over conducted between June and August 2016 by the Population Research Laboratory (CQUniversity) (23). Participants were recruited by a random digit dialling (both landlines and mobile lines) from a database (Sampleworx Pty Ltd) by random selection, with replacement, chosen by a computer program (23). Postcode was considered so that all respondents had an equal chance to be contacted (23). For landline phone numbers, the place of residence was pre-categorised as a male or female household to also allow for an equal chance to be contacted (23). If there was more than one male/female in the household, then the male/female with the most recent birthday was selected (23). If interviewers were not able to obtain a response from a household, a further five call-back attempts were made prior to moving onto the next random number in the database (23). The inclusion criteria were as follows: 18 years and over, were living in Australia and could be contacted by telephone (23).

### Survey instrument and methodology

A computer-assisted telephone interviewing (CATI) survey consisting of 160 questions was administered and took participants an average of 42 min to complete (23). The CATI comprised three blocks of questions: Core demographic questions and core health questions drawn from previous Queensland Social Survey questionnaires (24) and other research-related questions from additional organisations (23). In this paper, we only describe survey questions as relevant to our study aim. These are included in Additional File 1. A script was followed and included a brief standardised introduction followed by the three question blocks (23). The survey was pilot tested by trained interviewers in 40 randomly selected households (23). Minor adjustments were made regarding inadequate response categories, improving wording and question order following the pilot testing (23). Participant responses were directly entered into a database during the CATI (23). Quality assurance testing (random sample of 10%) indicated that the interviews were of high quality (mean score of 8.6 out of 10), the performance of interviewers was highly rated (mean score of 9.5 out of 10) and the questions were easy to understand (81% scored mostly easy or very easy)(23).

### Survey data

The CATI survey collected the following self-reported socio-demographic data: Age, sex, language spoken at home, residential postcode, and educational attainment (23). Residential postcode was used to determine participant geographic locale (city, rural, town) and estimate participant level of disadvantage by the Socioeconomic Index for Areas (SEIFA) Index of Relative Socioeconomic Advantage and Disadvantage (IRSAD) (25). Self-reported data on height (cm), weight (kg) and chronic health conditions were also collected. Height and weight were used to calculate body mass index (BMI; kg/m^2^), and participants were then categorised as underweight (< 18.5 kg/m^2^), normal weight (18.5–24.9 kg/m^2^), and overweight/obese (≥ 25 kg/m^2^) (26).

Within the core health questions, participants were asked how many serves of fruit and vegetables they eat on a usual day. A serve was defined in the question as being one medium piece or two small pieces of fruit (excludes dried fruit and fruit juice) and half a cup of cooked vegetables or one cup of salad vegetables (24, 27). Within the research-related questions, participants were asked five questions pertained to salt-related KABs, including the main source of salt in Australian diets, perceived salt intake compared to recommendations, frequency of added salt during cooking and placing a salt shaker on the table, and whether they are trying to cut down on salt intake (Additional File 1).

### Data analysis

Data were analysed using Stata v15.0 (StataCorp LLC, Texas, USA). To correct for over- or under-representation of certain groups in the Australian population, Population Research Laboratory (CQUniversity) created a weighting variable using age, sex and state location data from the Australian Bureau of Statistics (23). All data presented are weighted data.

Descriptive statistics (mean, 95% confidence interval, %) for socio-demographic factors were calculated. Selected variables were collapsed into dichotomous or trichotomous variables for further analysis, including age (18–44 years, 45 years and older), education (secondary schooling or under, technical or further education, university or higher education), and weight status category using BMI (underweight, healthy weight, overweight and obese). Responses to salt-related questions were dichotomised: main source of salt (correctly identified processed foods vs incorrect response), perceived salt consumption (more than recommended vs less than recommended, about the right amount, I don’t know), and salt use behaviour questions (always/often/sometimes vs rarely/never). To enable comparison of survey data to the Australian Dietary Guidelines (27), variables were created for compliance to fruit guidelines (< 2 serves, ≥ 2 serves) and vegetable guidelines (< 5 serves, ≥ 5 serves).

Weighted-adjusted logistic regression models were used to determine the differences in salt behaviours and fruit and vegetable consumption by socio-demographic factors. The socio-demographic factors included in the models were: sex, age group, geographic locale, education level, language spoken at home, weight category, and SEIFA quintile. One model was fitted for each of 10 outcome measures, and the adjusted proportions (or means) were derived for each socio-demographic factor post-estimation (using the margins command in Stata). A *p*-value of < 0.05 was considered statistically significant.

### Ethical approval

Ethical approval for this study was obtained from Deakin University Human Ethics Advisory Group – Health (project: 71_2016, Monitoring Salt Intake in Victoria). The 2016 National Social Survey received approval by the Human Ethics Research Review Panel at CQUniversity (project: H14/09–203, NATIONAL SOCIAL SURVEY 2016).

## Results

### Sample characteristics

A total of 1217 participants completed the CATI, 51% were female. The response rate (proportion of individuals participating / number of individuals invited) was 26% (23). More than half the sample were currently residing in cities (54%) and from either NSW (32%) or Victoria (25%). Over 70% of participants had completed an educational qualification higher than high school. Most participants spoke English at home (79.6%). More than half of participants were overweight or obese (56.4%; Table 1).

**Table 1:** Sociodemographic characteristics of weighted sample, by sex

	**Total**	**Males**	**Females**
**Sex** (%, 95% CI)		49.0 (45.8–52.2)	51.0 (47.8–54.2)
**Age category (%, 95% CI)**			
18–24 years	12.2 (9.8–15.0)	12.8 (9.3–17.3)	11.6 (8.7–15.4)
25–34 years	18.9 (16.2–22.1)	19.3 (15.3–24.0)	18.6 (14.9–23.0)
25–44 years	17.7 (15.3–20.2)	17.8 (14.6–21.7)	17.5 (14.3–21.1)
45–54 years	17.2 (15.0–19.7)	17.3 (14.1–21.0)	17.2 (14.2–20.7)
55–64 years	14.9 (13.1–16.8)	14.8 (12.3–17.7)	14.9 (12.6–17.6)
65 years and over	19.1 (17.2–21.3)	18.0 (15.4–21.0)	20.2 (17.3–23.4)
**State or territory currently residing in** (%, 95% CI)			
ACT	1.5 (1.0–2.3)	1.7 (1.1–2.6)	1.3 (0.6–2.9)
NSW	32.3 (29.4–35.3)	32.2 (28.1–36.6)	32.3 (28.4–36.5)
NT	0.5 (0.2–1.1)	0.6 (0.2–1.8)	0.5 (0.2–1.2)
QLD	20.0 (17.7–22.4)	20.0 (16.8–23.5)	20.0 (16.9–23.5)
SA	7.3 (5.7–9.3)	7.3 (5.1–10.3)	7.3 (5.1–10.2)
TAS	2.1 (1.3–3.5)	1.9 (0.9–4.1)	2.4 (1.3–4.4)
VIC	25.3 (22.8–28.1)	25.2 (21.6–29.2)	25.4 (21.9–29.3)
WA	11.0 (8.6–13.9)	11.2 (7.6–16.3)	10.8 (8.1–14.3)
**Geographic locale** (%, 95% CI)			
City	53.6 (50.3–56.8)	53.8 (49.0–58.5)	53.3 (48.9–57.7)
Town	23.4 (20.6–26.5)	22.8 (18.6–27.7)	24.0 (20.5–28.0)
Rural	23.0 (20.6–25.7)	23.4 (19.9–27.3)	22.6 (19.3–26.4)
**SEIFA (IRSAD) quintiles** (%, 95% CI)			
Quintile 1 (most disadvantaged)	13.8 (11.8–16.1)	12.3 (9.8–15.4)	15.2 (12.3–18.7)
Quintile 2	17.6 (15.3–20.1)	19.0 (15.8–22.8)	16.2 (13.2–19.7)
Quintile 3	19.3 (16.8–22.0)	19.7 (16.1–23.9)	18.9 (15.6–22.6)
Quintile 4	20.3 (17.6–23.2)	18.9 (15.0–23.5)	21.6 (18.2–25.5)
Quintile 5 (most advantaged)	29.0 (26.2–32.1)	30.0 (26.0–34.4)	28.1 (24.2–32.3)
**Highest level of education** (%, 95% CI)			
Secondary school or under	26.9 (24.3–29.8)	27.0 (23.2–31.1)	26.9 (23.3–30.9)
Technical or further education (TAFE)	22.3 (19.9–24.9)	24.6 (21.0–28.5)	20.1 (17.0–23.7)
University or higher education	50.8 (47.5–54.0)	48.5 (43.8–53.2)	53.0 (48.6–57.3)
**Speaks a language other than English at home** (%, 95% CI)	20.4 (17.8–23.2)	20.7 (17.1–24.8)	20.1 (16.6–24.1)
**Weight category** (%, 95% CI)			
Underweight	2.8 (1.7–4.5)	2.5 (1.1–5.4)	3.0 (1.6–5.7)
Healthy weight	40.8 (37.7–44.0)	37.7 (33.2–42.5)	43.8 (39.5–48.2)
Overweight/obese	56.4 (53.2–59.6)	59.8 (55.0–64.4)	53.1 (48.7–57.5)

*SEIFA* Socio-Economic Indexes for Areas, *IRSAD* Index of Relative Socio-Economic Advantage and Disadvantage

### Fruit and vegetable consumption

**Fig. 1:**
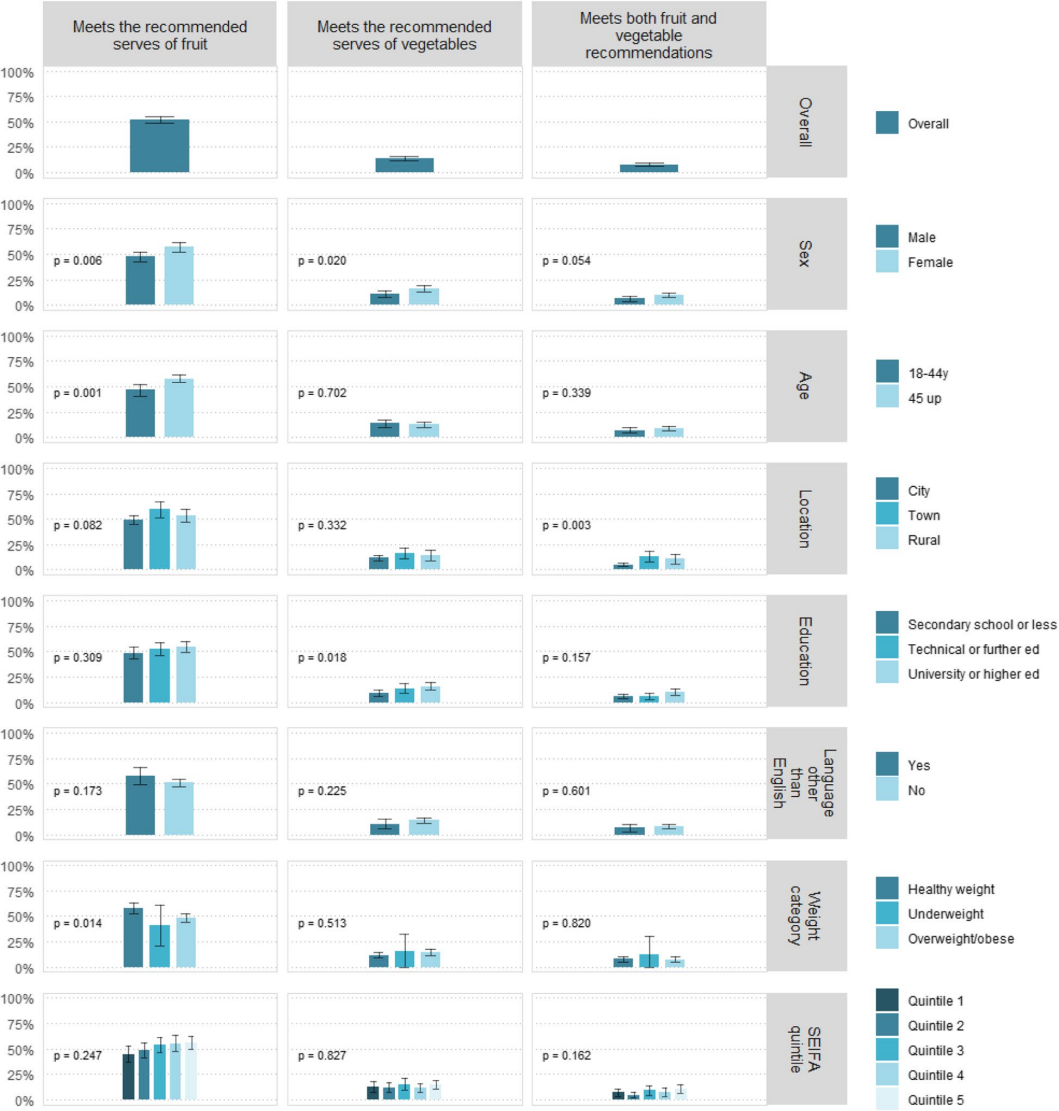
Fruit and vegetable consumption, overall and by socio-demographic variable

Less than 8% of participants reported consuming the recommended 2 or more serves of fruit and 5 or more serves of vegetables. Just over half reported meeting the fruit recommendation and only 13% meeting the vegetable recommendation (Fig. 1, Additional File 2).

There was no consistent pattern between meeting fruit and vegetable recommendations and socio-demographic factors. Meeting both fruit and vegetable recommendations only differed by geographic locale (*p* = 0.003), with a much lower proportion of participants living in city areas reporting adequate serves. Meeting fruit recommendations and meeting vegetable recommendations alone differed by sex (*p* = 0.006 and *p* = 0.020, respectively), with a higher proportion of females meeting each recommendation. A higher proportion of older and healthy weight participants reported meeting the fruit guidelines (*p* = 0.001 and *p* = 0.014, respectively), and the proportion of participants meeting the vegetable recommendation was higher in more educated sub-groups (*p* = 0.018; Fig. 1, Additional File 2).

### Salt-related knowledge, attitudes and behaviours

**Fig. 2:**
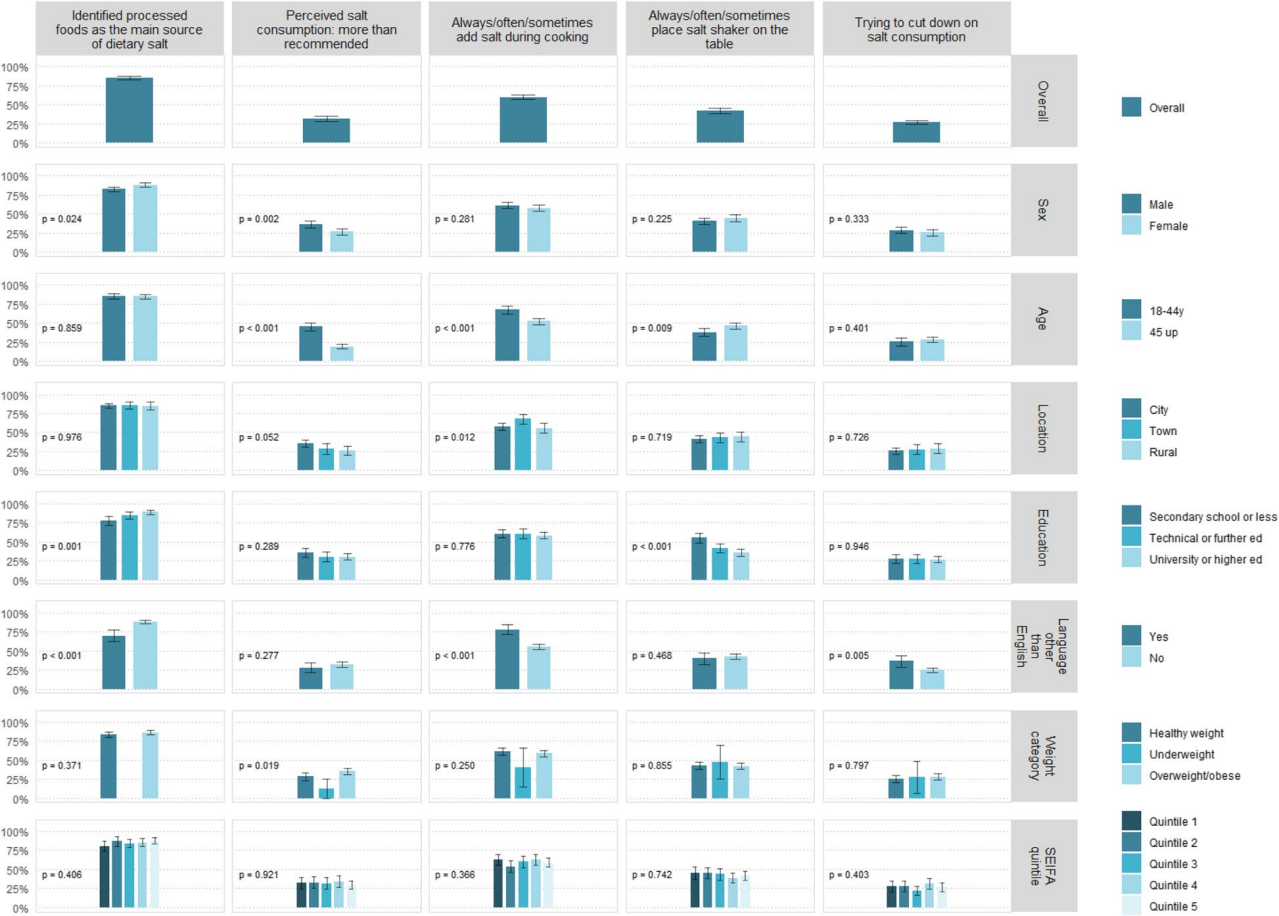
Salt knowledge, attitudes and behaviours, overall and by sociodemographic variable

Eighty-five per cent of participants knew that processed foods are the main source of salt in the Australian diet and about one-third perceived their daily salt intake to be “more than recommended”. Around a quarter reported actively trying to cut down on salt intake. Almost 60% of participants frequently (always/often/sometimes) added salt during cooking/meal preparation and 42% of respondents frequently placed a salt-shaker on the table at mealtimes (Fig. 2, Additional File 2).

There was no consistent pattern between salt KABs and socio-demographic factors. A higher proportion of participants that were female (*p* = 0.024), more highly educated (*p* = 0.001) and spoke English at home (*p* < 0.001) responded correctly to the knowledge question. The proportion of participants that perceived their salt intake to be more than the recommended amount was higher in males (*p* = 0.002), younger (*p* < 0.001) and overweight/obese participants (*p* = 0.019; Fig. 2, Additional File 2).

For salt behaviours, a higher proportion of participants speaking a language other than English at home reported taking action to reduce salt intake (*p* = 0.005). A higher proportion of participants that were younger (*p* < 0.001), lived in towns (*p* = 0.012), and spoke a language other than English at home (*p* < 0.001) frequently added salt while cooking, yet a higher proportion of older (*p* = 0.009) and less educated participants (*p* < 0.001) placed a salt shaker on the table (Fig. 2, Additional File 2).

## Discussion

This study found no consistent pattern between socio-demographic factors and fruit and vegetable consumption and salt behaviours in a large, nationally representative sample of Australian adults. Our results confirmed that fruit and vegetable consumption and salt behaviours in Australia are poor across the adult population. As such, broad population-based policies and programs to improve fruit and vegetable intake and salt behaviours are needed. This could include a nationwide consumer awareness campaign on salt, fruit and vegetables to stimulate consumer behaviour change to improve Australian’s diets.

The overall salt KAB results were consistent with past research in national and sub-national samples of Australians. The majority of participants knew processed foods were the main source of salt in the Australian diet (14, 18, 22). However, most reported frequent use of discretionary salt (10, 14–19, 21) and very few reported salt reduction actions being taken (18). These findings reinforce that Australians perform adverse salt behaviours and highlight an important opportunity for consumer education. Consumer education campaigns have been effective in reducing discretionary salt consumption at the population level (28) and a national campaign focusing on reducing discretionary salt intake could stimulate individual action to reduce salt intake and be effective in reducing salt intake by up to 25% (29). At present, only around one-quarter of participants are actively trying to reduce their salt intake. This is a much lower proportion than identified in previous sub-national samples (18, 19), which may reflect current consumer priorities of eating more fruit and vegetables, and less sugar and fat, before reducing salt intake (30). The low proportion of consumers currently trying to cut down on salt illustrates the potential for reducing salt intake through consumer awareness and behaviour change strategies.

Similarly, fruit and vegetable findings were consistent with previous national surveys. Around half of participants reported meeting the fruit guidelines and around 10% reported meeting the vegetable guidelines, while an even smaller proportion reported meeting both guidelines (2, 10, 11). These results reinforce the poor fruit and vegetable intakes of Australian’s and highlight the scope for consumer awareness and behaviour change strategies to improve nationwide consumption.

Differences between the findings in this study compared to non-nationally representative samples highlight the need to undertake this research to understand whether socio-demographic factors are related to salt behaviours and fruit and vegetable intake in distinct populations. There was no consistent pattern between socio-demographic factors and fruit and vegetable consumption and salt behaviours in the current nationally-representative sample. The few socio-demographic differences that were identified were mostly dissimilar to those found in other samples (12, 18). For example, sex was not associated with discretionary salt behaviours, and was associated with attitudes regarding personal salt consumption in the current study, while the converse was true in the Victorian population (18). Also, a higher proportion of females reported consuming adequate fruit and vegetable intakes compared to males in this study, while Nour et al. determined males consumed more fruit than females and there was no difference in vegetable intake in young adults (12). These inconsistencies likely reflect the different population samples and highlight the importance of understanding if there are socio-demographic differences in a specific target population when designing consumer awareness and behaviour change strategies.

A major difference between the current study and Nour et al.’s young adults’ study was the proportion meeting the fruit and vegetable guidelines (12). Around 15% of young adults (18–34 years) met fruit and vegetable guidelines (12), compared to just 7% in the current study (18–44 years). This disparity is likely due to the different dietary methodologies used. Nour et al. conducted a secondary analysis of 24-h recall data, a subset of the Australian Health Survey data, to calculate intake of fruit and vegetables in grams per day and convert this to serves per day (12). While, in the main analysis of the Australian Health Survey and the current study, self-reported serves per day was the measure used (10). This suggests that Australian’s underestimate the number of serves of fruits and vegetables consumed per day compared to calculations from dietary recall data. This finding illustrates that self-reported serves per day may not be a reliable indicator of fruit and vegetable intake. Since this method is commonly used in population studies, for example, the World Health Organization STEPwise approach to surveillance survey (31), further research assessing the accuracy of this method is warranted.

This was the first study to investigate socio-demographic differences in measures related to salt, fruit and vegetable intake in a random and nationally representative survey sample. It allows understanding of baseline measures related to salt, fruit and vegetable intake in the population and population subgroups, which can be used to inform broad population-based nutrition policies and programs. The study benefited from a rigorous CATI protocol and interviewer training. However, some limitations should be noted. Despite a sampling strategy aiming to be reflective of the national population and the use of an adjustment weight, the survey response rate was around one-quarter, which is low compared to the response rates of the national health surveys (2, 10, 11), and caution should be used in generalizing the findings to the Australian population. The National Social Survey was a lengthy self-reported questionnaire, which may be subject to participant biases (e.g. social desirability bias) and may lead to participant and interviewer fatigue. The study assessed salt knowledge, attitudes and behaviours but did not assess salt intake as self-reported dietary assessment methods are not a reliable indicator of salt intake. Further to this, the findings suggest self-reported serves may not be a reliable indicator of fruit and vegetable intake, which should be considered in future surveys. In addition, adherence to vegetable recommendations was likely over-estimated. For this analysis, we used 5 serves per day as the recommendation for the whole population, similar to other studies in this area (12), although we note the recommended number of serves of vegetables for males aged 19–50 is 6 serves per day and for males aged 51–70 is 5.5 serves per day (27). The questionnaire only included five salt questions and two fruit and vegetable questions, so while adding to the evidence base, more comprehensive surveys are required to fully understand the impact of socio-demographic variables on dietary factors including fruit, vegetable and salt intakes.

## Conclusion

There was no consistent pattern between socio-demographic factors and salt behaviours and fruit and vegetable consumption. The frequent use of discretionary salt and low indication of individuals taking action to reduce salt intake, alongside less than recommended intakes of fruit and vegetable, are placing Australians at risk of diet-related NCDs. As such, broad population-based policies and programs to improve fruit and vegetable intake and salt behaviours are needed. This could include a nationwide consumer awareness campaign to stimulate consumer behaviour change and policies to improve the food environment to support consumers to change their behaviours to improve Australian’s diets.

## Supplementary Information

**Additional file 1.**

**Additional file 2.**

**Additional file 3.**

## Data Availability

The datasets used and/or analysed during the current study are available from the corresponding author on reasonable request.

## Abbreviations

BMI: Body mass index
CATI: Computer-assisted telephone interviewing
CVD: Cardiovascular disease
DALY: Disability-adjusted life year
IRSAD: Index of Relative Socioeconomic Advantage and Disadvantage
KAB: Knowledge, attitudes and behaviours
NCD: Non-communicable disease
SEIFA: Socioeconomic Index for Areas

## References

[1] Institute for Health Metrics and Evaluation (IHME). GBD Compare Data Visualization Seattle, WA: IHME, University of Washington; 2016 [Available from: https://vizhub.healthdata.org/gbd-compare/.

[2] Australian Bureau of Statistics. 4364.0.55.001 - National Health Survey: First Results Australia 2017–18 Canberra: Australian Bureau of Statistics; 2018 [Available from: https://www.ausstats.abs.gov.au/ausstats/subscriber.nsf/0/4B3976684C09F43FCA258399001CE630/$File/4364.0.55.001%20-%20national%20health%20survey,%20first%20results,%202017-18.pdf.

[3] Land M-A, Nowson CA, Petersen KS, Margerison C, Neal BC, Johnson C (2018). Salt consumption by Australian adults: a systematic review and meta-analysis. Med J Aust.

[4] World Health Organization. Prevention of cardiovascular disease: guidelines for assessment and management of cardiovascular risk Geneva: World Health Organization; 2007 [Available from: https://apps.who.int/iris/handle/10665/43685.

[5] Graudal NA, Hubeck-Graudal T, Jürgens G. Effects of Low-Sodium Diet vs. High-Sodium Diet on Blood Pressure, Renin, Aldosterone, Catecholamines, Cholesterol, and Triglyceride (Cochrane Review). Am J of Hypertens. 2012;25(1):1–15. 10.1038/ajh.2011.210 22068710

[6] World Health Organization (2013). Global Action Plan for the Prevention and Control of Noncommunicable Diseases 2013–2020.

[7] World Health Organization (2016). Report on the Commission on Ending Childhood Obesity Geneva.

[8] Sutherland J, Edwards P, Shankar B, Dangour AD (2013). Fewer adults add salt at the table after initiation of a national salt campaign in the UK: a repeated cross-sectional analysis. Br J Nutr.

[9] Khokhar D, Nowson CA, Margerison C, Bolam B, Grimes CA (2018). Knowledge and attitudes are related to selected salt-specific behaviours among Australian parents. Nutrients.

[10] Australian Bureau of Statistics. 4364.0.55.001 - Australian Health Survey: First Results, 2011–12 Canberra: Australian Bureau of Statistics; 2011 [Available from: https://www.ausstats.abs.gov.au/ausstats/subscriber.nsf/0/1680ECA402368CCFCA257AC90015AA4E/$File/4364.0.55.001.pdf.

[11] Australian Bureau of Statistics. 4364.0.55.001 - National Health Survey: First Results Australia 2014–15 Canberra: Australian Bureau of Statistics; 2015 [Available from: https://www.ausstats.abs.gov.au/ausstats/subscriber.nsf/0/CDA852A349B4CEE6CA257F150009FC53/$File/national%20health%20survey%20first%20results,%202014-15.pdf.

[12] Nour M, Sui Z, Grech A, Rangan A, McGeechan K, Allman-Farinelli M (2017). The fruit and vegetable intake of young Australian adults: a population perspective. Public Health Nutr.

[13] Livingstone KM, Olstad DL, Leech RM, Ball K, Meertens B, Potter J (2017). Socioeconomic inequities in diet quality and nutrient intakes among Australian adults: findings from a nationally representative cross-sectional study. Nutrients.

[14] Charlton K, Yeatman H, Houweling F, Guenon S (2010). Urinary sodium excretion, dietary sources of sodium intake and knowledge and practices around salt use in a group of healthy Australian women. Aust N Z J Public Health.

[15] Grimes C, Riddell L, Nowson C (2010). The use of table and cooking salt in a sample of Australian adults. Asia Pac J Clin Nutr.

[16] Nowson C, Lim K, Grimes C, O’Halloran S, Land MA, Webster J, et al. Dietary Salt Intake and Discretionary Salt Use in Two General Population Samples in Australia: 2011 and 2014. Nutrients. 2015;7(12):10501–12. 10.3390/nu7125545 PMC4690097 26694459

[17] Sarmugam R, Worsley A, Wang W (2013). An examination of the mediating role of salt knowledge and beliefs on the relationship between socio-demographic factors and discretionary salt use: a cross-sectional study. Int J Behav Nutr Phys Act.

[18] Grimes CA, Kelley S-J, Stanley S, Bolam B, Webster J, Khokhar D (2017). Knowledge, attitudes and behaviours related to dietary salt among adults in the state of Victoria, Australia 2015. BMC Public Health.

[19] Land M-A, Webster J, Christoforou A, Johnson C, Trevena H, Hodgins F (2014). The association of knowledge, attitudes and behaviours related to salt with 24-hour urinary sodium excretion. Int J Behav Nutr Phys Act.

[20] Grimes CA, Riddell LJ, Nowson CA (2009). Consumer knowledge and attitudes to salt intake and labelled salt information. Appetite.

[21] O'Reilly S, Brinkman M, Giles G, English D, Nowson C. Sodium intake and use of discretionary salt in an Australian population sample. Proc Nutr Soc. 2010;69(OCE1).

[22] Webster J, Li N, Dunford EK, Nowson CA, Neal B (2010). Consumer awareness and self-reported behaviours related to salt consumption in Australia. Asia Pac J Clin Nutr.

[23] CQUniversity Australia Population Research Laboratory. 2016 National Social Survey. Final Technical & Sampling Report. Rockhampton, Queensland: CQUniversity Australia; 2016.

[24] Queensland Government Statistician’s Office, Queensland Treasury. Queensland Social Survey 2018, Social Cohesion Survey Report: The State of Queensland (Queensland Treasury) 2018 [Available from: https://www.qgso.qld.gov.au/issues/2831/social-cohesion-qld-social-survey-report-2018.pdf.

[25] Australian Bureau of Statistics. 2033.0.55.001 - Technical Paper. Socio-Economic Indexes for Areas (SEIFA) Canberra: Australian Bureau of Statistics; 2016 [Available from: https://www.ausstats.abs.gov.au/ausstats/subscriber.nsf/0/756EE3DBEFA869EFCA258259000BA746/$File/SEIFA%202016%20Technical%20Paper.pdf.

[26] World Health Organization Regional Office for Europe. Body mass index - BMI Copenhagen, Denmark: World Health Organization; 2020 [Available from: http://www.euro.who.int/en/health-topics/disease-prevention/nutrition/a-healthy-lifestyle/body-mass-index-bmi.

[27] National Health and Medical Research Council. Australian Dietary Guidelines Canberra: National Health and Medical Research Council; 2013 [Available from: https://www.health.gov.au/sites/default/files/australian-dietary-guidelines.pdf.

[28] Ide N, Ajenikoko A, Steele L, Cohn J, J Curtis C, Frieden TR, et al. Priority Actions to Advance Population Sodium Reduction. Nutrients. 2020;12(9):2543. 10.3390/nu12092543 PMC7551205 32842580

[29] Bhat S, Marklund M, Henry ME, Appel LJ, Croft KD, Neal B (2020). A systematic review of the sources of dietary salt around the world. Adv Nutr.

[30] IPSOS. Food CHATs. Australia's most comprehensive study integrating consumer attitudinal trends with consumption behaviour change Australia: IPSOS; 2016 [Available from: https://www.ipsos.com/sites/default/files/2016-06/023.1-Food-in-Australia.pdf.

[31] World Health Organization (2016). WHO STEPS Instrument (Core and Expanded) Geneva.

