# Supplementary material for: Are there socio-demographic differences in salt behaviours and fruit and vegetable consumption in Australian adults? A nationally representative cross-sectional survey
Source: Nutr J. 2021 Sep 8;20:77. doi: 10.1186/s12937-021-00734-0 (PMC8425065; doi:10.1186/s12937-021-00734-0)
Supplement: Supplementary file 1 — Additional file 1. [file 12937_2021_734_MOESM1_ESM.docx]

Additional file 1. Excerpt of the 2016 National Social Survey Questionnaire

**Demographic Questions**

Record respondent's gender (ask only if necessary)

1. Male
2. Female

What is your current age?

[Enter age in years]

Can I confirm the state or territory that you are currently residing in?

1. Australian Capital Territory (ACT)
2. New South Wales (NSW)
3. Northern Territory (NT)
4. Queensland (QLD)
5. South Australia (SA)
6. Tasmania (TAS)
7. Victoria (VIC)
8. Western Australia (WA)
9. No response

Do you presently live in a city, town, or rural area?

1. City
2. Town
3. Rural area
4. (Do not read) Don't know/No response

For an accurate idea of the geographic spread of the respondents, could you please tell me your postcode?

[Enter postcode]

What is your highest level of education? This includes completed or incomplete.

1. Pre-school
2. Infants/primary school
3. Secondary/high school
4. Technical or further educational institution (inc TAFE colleges)
5. University or other higher educational institution
6. None
7. (Do not read) Don't know/No response

Do you speak a language other than English at home?

1. Yes
2. No
3. (Do not read) Don't know
4. (Do not read) No response

Would you describe yourself as:

1. Aboriginal or
2. Torres Strait Islander
3. Australian South Sea Islander
4. None of the above
5. (Do not read) Don't know
6. (Do not read) No response

How tall are you in centimetres?

[Enter height in centimetres]

What is your weight in kilograms?

[Enter weight in kilograms]

**Self-reported chronic health problems**

Have you ever been told by a doctor that you have any chronic health problems, including the following: (read list and select all that apply)

1. Heart disease
2. High blood pressure
3. Stroke
4. Cancer
5. Depression/Anxiety
6. Diabetes Type 1
7. Diabetes Type 2
8. Arthritis
9. Chronic back/neck pain
10. Asthma
11. COPD (airways disease, emphysema)
12. Chronic kidney/renal disease
13. None of the above

**Fruit and Vegetable Consumption: NSS Core Questions**

Ok now we will ask you about some of your current health behaviours.

How many serves of vegetables do you eat on a usual day? One serve of vegetables is equivalent to half a cup of cooked vegetables or one cup of salad vegetables.

[Enter number of serves per day, 0 for none, 99 for no response]

How many serves of fruit do you eat on a usual day? One serve of fruit is equivalent to one medium piece or two small pieces of fruit.

[Enter number of serves per day, 0 for none, 99 for no response]

**Salt Consumption: Deakin University School of Exercise and Nutrition Sciences (Sponsor)**

Which of the following do you think is the main source of salt in the Australian diet?

1. Salt added during cooking or at the table
2. Salt from processed foods such as breads, sausages and cheese
3. Salt from natural food sources
4. (Do not read) Don't know/Unsure
5. (Do not read) No response

How do you think your daily salt intake compares to the amount of salt recommended by health professionals?

1. I eat less salt than recommended
2. I eat about the right amount of salt
3. I eat more salt than recommended
4. (Do not read) Don't know/Unsure
5. (Do not read) No response

In the food you eat at home, how often is salt added during cooking?

1. Always
2. Often
3. Sometimes
4. Rarely
5. Never
6. (Do not read) Don't know/Unsure
7. (Do not read) No response

Do you place a salt shaker on your table at meal times?

1. Always
2. Often
3. Sometimes
4. Rarely
5. Never
6. (Do not read) Don't know/Unsure
7. (Do not read) No response

Are you trying to cut down on the amount of salt you eat?

1. Yes
2. No
3. (Do not read) Don't know/Unsure
4. (Do not read) No response
