# Supplementary material for: Are there socio-demographic differences in salt behaviours and fruit and vegetable consumption in Australian adults? A nationally representative cross-sectional survey
Source: Nutr J. 2021 Sep 8;20:77. doi: 10.1186/s12937-021-00734-0 (PMC8425065; doi:10.1186/s12937-021-00734-0)
Supplement: Supplementary file 2 — Additional file 2. [file 12937_2021_734_MOESM2_ESM.docx]

Additional File 2

Supplementary Table. Fruit and vegetable consumption and salt knowledge, attitudes and behaviours, overall and by sociodemographic variable

|  | **FRUIT AND VEGETABLE CONSUMPTION** | | | **SALT KNOWLEDGE, ATTITUDES AND BEHAVIOURS** | | | | |
| --- | --- | --- | --- | --- | --- | --- | --- | --- |
|  | **Meets the recommended serves of fruit**  (%, 95% CI) | **Meets the recommended serves of vegetables**  (%, 95% CI) | **Meets both fruit and vegetable recommendations**  (%, 95% CI) | **Identified processed foods as the main source of salt in the diet**^[[1]](#footnote-1)^  (%, 95% CI) | **Perceived salt consumption: more than recommended**  (%, 95% CI) | **Always/often/ sometimes add salt during cooking**  (%, 95% CI) | **Always/often/ sometimes place a salt shaker on the table**  (%, 95% CI) | **Trying to cut down on salt consumption**  (%, 95% CI) |
| **OVERALL** | 52.4 (49.1-55.6) | 13.4 (11.3-15.7) | 7.9 (6.3-9.7) | 84.9 (82.5-87) | 31.8 (28.6-35.1) | 59.7 (56.6-62.8) | 42.2 (39.1-45.4) | 26.8 (24-29.7) |
| **Sex** |  |  |  |  |  |  |  |  |
| Male | 47.7 (42.8-52.6) | 10.6 (7.6-13.5) | 6.1 (3.7-8.4) | 82.4 (79.1-85.7) | 36.7 (32-41.4) | 61 (56.8-65.2) | 40.6 (36.3-44.9) | 28.4 (24.1-32.6) |
| Female | 57.0 (52.6-61.4) | 15.9 (12.7-19.1) | 9.5 (7.1-12) | 87.7 (84.7-90.8) | 26.6 (22.6-30.7) | 57.7 (53.4-61.9) | 44.4 (40-48.7) | 25.5 (21.7-29.4) |
| p-value | **0.006** | **0.02** | 0.054 | **0.024** | **0.002** | 0.281 | 0.225 | 0.333 |
| **Age** |  |  |  |  |  |  |  |  |
| 18-44 years | 46.4 (40.9-51.8) | 13.7 (10-17.4) | 7.0 (4.3-9.7) | 85.3 (81.4-89.3) | 45.4 (39.8-51) | 67.5 (62.3-72.6) | 37.7 (32.5-42.9) | 25.5 (20.7-30.3) |
| 45 years and over | 57.8 (53.9-61.7) | 12.8 (10.3-15.3) | 8.8 (6.6-10.9) | 84.8 (82-87.7) | 19.2 (16.2-22.3) | 52.4 (48.5-56.3) | 46.8 (42.8-50.8) | 28.3 (24.6-32) |
| p-value | **0.001** | 0.702 | 0.339 | 0.859 | **<0.001** | **<0.001** | **0.009** | 0.401 |
| **Geographic location** |  |  |  |  |  |  |  |  |
| City | 48.8 (44.5-53.2) | 11.7 (8.8-14.6) | 5.0 (3.2-6.7) | 85.1 (81.9-88.3) | 35.3 (31.1-39.6) | 57.7 (53.3-62) | 41.3 (37-45.7) | 25.9 (22-29.7) |
| Town | 59.5 (51.7-67.3) | 16.3 (10.8-21.8) | 13.5 (8-18.9) | 85.4 (81.1-89.8) | 28.1 (21-35.2) | 67.6 (61.5-73.8) | 43 (36.3-49.6) | 27.5 (21-34) |
| Rural | 53.4 (46.8-60) | 14.1 (9.2-19.1) | 10.5 (6-15) | 84.7 (79.7-89.7) | 26 (19.9-32.2) | 55.3 (48.8-61.7) | 44.6 (38.2-51.1) | 28.9 (22.7-35.1) |
| p-value | 0.082 | 0.332 | **0.003** | 0.976 | 0.052 | **0.012** | 0.719 | 0.726 |
| **Education level** |  |  |  |  |  |  |  |  |
| Secondary school or less | 48.5 (42.6-54.4) | 8.9 (6-11.8) | 6.0 (3.6-8.4) | 77.5 (72.2-82.9) | 35.7 (29.7-41.7) | 60.5 (55-66) | 55 (48.7-61.4) | 27.7 (21.9-33.5) |
| Technical or further ed | 52.0 (45.7-58.4) | 13.6 (9.1-18.1) | 6.1 (2.8-9.4) | 84.3 (79.6-89) | 30 (23.8-36.2) | 60.3 (54.3-66.4) | 41.4 (35.2-47.6) | 27.1 (21.3-32.9) |
| University or higher ed | 54.7 (49.7-59.6) | 15.7 (12-19.3) | 9.8 (6.8-12.9) | 88.9 (86-91.9) | 30.2 (25.8-34.7) | 58.1 (53.6-62.6) | 35.9 (31.4-40.4) | 26.5 (22.3-30.6) |
| p-value | 0.309 | **0.018** | 0.157 | **0.001** | 0.289 | 0.776 | **<0.001** | 0.946 |
| **Speak a language other than English at home** |  |  |  |  |  |  |  |  |
| Yes | 51.0 (47.2-54.9) | 14.0 (11.4-16.6) | 8.1 (6.1-10.1) | 88.3 (86-90.5) | 32.5 (28.9-36.1) | 55.2 (51.7-58.8) | 43.1 (39.7-46.5) | 24.7 (21.6-27.8) |
| No | 57.9 (49.5-66.3) | 10.3 (5.5-15) | 6.9 (3.1-10.6) | 69.8 (62.4-77.2) | 28 (21.2-34.8) | 77.8 (71.5-84.2) | 39.9 (32.3-47.5) | 36.4 (28.7-44.1) |
| p-value | 0.173 | 0.225 | 0.601 | **<0.001** | 0.277 | **<0.001** | 0.468 | **0.005** |
| **Weight category** |  |  |  |  |  |  |  |  |
| Healthy weight | 58.3 (53.1-63.5) | 11.7 (8.7-14.7) | 7.9 (5.5-10.3) | 83.8 (80.1-87.5) | 28.3 (23.5-33.1) | 61.6 (56.8-66.3) | 43 (38.3-47.8) | 25.7 (21.2-30.2) |
| Underweight | 40.8 (20.7-60.9) | 15.8 (0-32.9) | 12.4 (0-30.1) | (-) | 12.2 (0-25.3) | 40.4 (14.5-66.3) | 47.3 (25.1-69.5) | 27.5 (6.8-48.3) |
| Overweight/obese | 48.7 (44.3-53) | 14.3 (11-17.6) | 7.6 (5.2-10.1) | 86 (83.1-88.9) | 35.2 (30.9-39.5) | 58.7 (54.8-62.7) | 41.9 (37.8-46) | 27.8 (23.9-31.7) |
| p-value | **0.014** | 0.513 | 0.82 | 0.371 | **0.019** | 0.250 | 0.855 | 0.797 |
| **SEIFA quintiles** |  |  |  |  |  |  |  |  |
| Quintile 1 | 44.5 (36.4-52.7) | 12.6 (7.2-18) | 6.8 (3-10.6) | 80.6 (74.3-86.9) | 31.9 (24.1-39.7) | 62.9 (55.6-70.2) | 45.1 (36.9-53.3) | 27.8 (20.5-35) |
| Quintile 2 | 48.5 (41-56.1) | 11.9 (7-16.7) | 4.5 (2.1-6.9) | 86.6 (80.7-92.4) | 32.4 (24.9-40) | 53.5 (45.8-61.2) | 45.1 (37.7-52.6) | 27.3 (20.4-34.2) |
| Quintile 3 | 53.6 (46.2-60.9) | 14.9 (9.2-20.5) | 9.1 (4.3-13.8) | 83.6 (78.4-88.9) | 31.7 (24.5-38.9) | 59.8 (52.7-66.8) | 43.5 (36.3-50.8) | 21.8 (15.7-27.9) |
| Quintile 4 | 55.1 (47-63.1) | 11.4 (6.7-16.1) | 7.4 (3.4-11.4) | 85.2 (80.2-90.2) | 33.6 (26-41.2) | 62.7 (55.9-69.4) | 38.8 (32-45.6) | 31.3 (24.4-38.2) |
| Quintile 5 | 55.8 (49.5-62) | 14.6 (10-19.2) | 10.6 (6.6-14.6) | 87.4 (83.4-91.4) | 29.4 (24-34.8) | 58.6 (52.8-64.5) | 41.4 (35.4-47.5) | 26.8 (21.2-32.5) |
| p-value | 0.247 | 0.827 | 0.162 | 0.406 | 0.921 | 0.366 | 0.742 | 0.403 |

1. For this outcome, underweight participants were dropped from the model (this was done by the software since these participants *predicted success perfectly* i.e. all underweight participants had the same response for this outcome). [↑](#footnote-ref-1)
